# Access to Blood Glucose Testing in Peru: Who Is Getting Tested?

**DOI:** 10.3390/epidemiologia6020020

**Published:** 2025-05-03

**Authors:** Jamee Guerra Valencia, Akram Hernández-Vásquez, Carlos Rojas-Roque, Rodrigo Vargas-Fernández

**Affiliations:** 1 Facultad de Ciencias de la Salud, Universidad Privada del Norte, Lima 15314, Peru; jamee.guerra@upn.pe; 2 Centro de Excelencia en Investigaciones Económicas y Sociales en Salud, Vicerrectorado de Investigación, Universidad San Ignacio de Loyola, Lima 15024, Peru; 3 Centre for Health Economics, University of York, York YO10 5DD, UK; carlos.rojasroque@york.ac.uk; 4 Epidemiology and Health Economics Research (EHER), Universidad Científica del Sur, Lima 15067, Peru; jvargasf@cientifica.edu.pe

**Keywords:** glycemic control, socioeconomic disparities in health, healthcare disparities, cross-sectional studies, Peru

## Abstract

Background/Objectives: Significant disparities in access to diabetes screening persist, particularly among populations with limited healthcare access. We aimed to estimate the proportion of overweight-obese Peruvian adults who underwent blood glucose testing (BGT) in the past year and to analyse the socioeconomic and geographic inequalities associated with access to this preventive intervention. Methods: We conducted a cross-sectional study using data from the Demographic and Family Health Survey 2023. We included adults aged 35–70 years diagnosed with overweight or obesity, according to the United States Preventive Services Task Force screening recommendation. We used concentration curves (CC) and concentration indices (CI) to assess socioeconomic inequalities in BGT. BGT was ascertained using a self-reported question, while the wealth index was used as the variable to measure inequality. We also conducted a decomposition analysis to determine the relative contributions of covariates to socioeconomic inequalities in BGT. Results: A total of 9499 individuals were included in the analysis. A pro-rich concentration of BGT uptake was observed in CC and CI (0.2090; *p* < 0.001). Notably, a 27-point prevalence difference was reported between the lowest and highest wealth index. The decomposition analysis showed that higher education (+64%) and rural areas (+10.6%) were the main contributors to this pro-rich concentration. In contrast, secondary education (−4.7%) and female gender (−3.4%) reduced this pro-rich concentration. Conclusions: The results underscore the need for targeted strategies, such as enhancing healthcare infrastructure and implementing localized screening initiatives, to close the gap and address the burden of undiagnosed diabetes in high-risk populations.

## 1. Introduction

Type 2 diabetes mellitus (T2DM) is one of the leading causes of morbidity and mortality worldwide, causing severe vascular complications and reducing quality of life [1]. In 2021, over 500 million people had T2DM, contributing to more than 75 million disability-adjusted life years (DALYs) [1]. Between 1990 and 2021, the age-standardized prevalence of diabetes in Latin America increased by 44.5%, among the lowest regional changes. However, in Andean countries (Bolivia, Chile, Peru), it doubled, with projections indicating a 61.2% rise by 2050 [2]. Body mass index (BMI) is the primary risk factor for diabetes DALYs globally. In regions like Latin America, it contributes to more than 60% of the disease burden [2], with variations observed across geographical, sociodemographic, and biological factors. Latin Americans, including those in Andean countries, are at a heightened risk of developing T2DM due to, among other factors, a combination of genetic predispositions and environmental factors. These include the ongoing nutrition transition experienced in the region [3], which exacerbates the challenges of disease management [2,4].

Undiagnosed diabetes remains a major challenge. In 2021, an estimated 239.7 million adults aged 20–79 had undiagnosed diabetes, representing 44.7% of all cases [5]. This prevalence is particularly pronounced in low- and middle-income countries (LMICs), where nearly half of individuals with diabetes remain undiagnosed, reflecting significant barriers to early detection [6]. In Latin America, about one-third of adults with diabetes are undiagnosed, increasing the risk of preventable complications and disease progression [5]. These undetected cases highlight current healthcare systems’ limitations and emphasize the need for targeted interventions [5]. Screening for diabetes serves as a critical preventive tool, bridging the gap between undiagnosed cases and timely management. The U.S. Preventive Services Task Force (USPSTF) recommends blood glucose testing (BGT) for nonpregnant adults aged 35–70 with overweight or obesity every three years, urging earlier screening for populations with disproportionately high diabetes prevalence, including Latinos [7]. However, the existing health insurance programmes in Peru and many Latin American countries are heavily based on a biomedical curative approach [8], which underscores significant gaps in preventive healthcare policies for high-risk populations.

Significant disparities in access to diabetes screening persist, particularly among populations with limited healthcare access. Socioeconomic status, education level, geographic location, and gender influence access to preventive services, including BGT [9,10]. For instance, individuals in rural areas or with lower income are less likely to receive recommended screenings [11]. Additionally, women and ethnic minorities, including Latino populations, often face compounded barriers due to intersecting social and healthcare inequalities [10]. In Peru, where diabetes prevalence is rising [2], evidence on disparities in BGT access remains scarce, especially among at-risk groups such as adults with overweight and obesity. The objective of the study was to estimate the proportion of overweight-obese Peruvian adults who underwent BGT in the past year and to analyse the socioeconomic and geographic inequalities associated with access to this preventive intervention.

## 2. Materials and Methods

### 2.1. Design and Data

A cross-sectional analytical study was conducted using secondary data from the Demographic and Family Health Survey (ENDES) 2023, a nationally representative Peruvian survey conducted annually by the National Institute of Statistics and Informatics (INEI). The ENDES 2023 was conducted with a probabilistic, multistage, and stratified sampling design, which guarantees representativeness at the national, regional, and urban/rural levels. The survey collects demographic, socioeconomic, and health information, following standardized processes to ensure data quality and consistency. The data sheet, questionnaire, dictionary, manuals and databases are publicly available on the ENDES website https://proyectos.inei.gob.pe/endes/ (accessed on 12 December 2024) and INEI https://proyectos.inei.gob.pe/microdatos/ (accessed on 12 December 2024).

### 2.2. Study Population

The study population included adults aged 35 to 70 years diagnosed with overweight or obesity, in accordance with the USPSTF screening recommendations for this age group [7]. This age range was selected based on the 2021 USPSTF guideline, which advises routine screening for prediabetes and T2DM in adults within this range who are overweight or obese. The recommendation is based on moderate to high certainty that screening provides a moderate to substantial net benefit. It reflects evidence of increasing diabetes incidence from age 35 onward and the value of early detection in reducing complications. Although earlier screening may be considered for high-risk groups, including Latino populations, this is presented as a clinical consideration rather than a graded recommendation [7].

Overweight and obesity were defined using the BMI, which is calculated as weight in kilograms divided by the square of height in meters: body weight (kg)/[height (m)]^2^. The cutoff points used were: BMI ≥ 25 for adults up to 59 years of age and BMI ≥ 28 for adults aged 60 years and older, based on criteria proposed by the Peruvian Ministry of Health [12]. Furthermore, since the USPSTF recommendation is intended to screen for prediabetes and T2DM, only non-diabetic patients were included, as determined by self-report.

Of the 36,760 households surveyed in ENDES 2023, 9499 eligible participants met the inclusion criteria. Participants with missing information on key variables were excluded from the analysis.

### 2.3. Outcome

The outcome variable was whether a BGT had been performed in the last year (yes/no). It was considered affirmative if the participant answered “yes” to the following question: “In the last 12 months, has any doctor or other healthcare personnel measured your blood glucose or ‘sugar’ in your blood?” This question follows the time frame (last 12 months) and action (blood glucose testing) structure of the WHO STEPwise approach to NCD risk factor surveillance (STEPS) question [13], but differs in specifying who performs the action.

### 2.4. Exposure

As the exposure variable, a wealth index was employed. This index, a composite measure of household wealth, was constructed using principal component analysis (PCA) applied to a set of variables encompassing household assets, access to services, and housing characteristics [14]. To facilitate population stratification and descriptive analysis, the derived wealth index was subsequently categorized into quintiles (I to V), ranging from the first quintile (lowest socioeconomic level) to the fifth quintile (highest socioeconomic level).

### 2.5. Covariates

The covariates or stratification variables include sociodemographic, economic, and geographic factors, selected based on the PROGRESS-Plus model, which addresses the multiple characteristics and data presentation in the study of inequalities [15]. These variables were as follows: gender (male, female), age groups (35–49, 50–59, 60–70 years), education level (up to primary, secondary, higher education), disability (no, yes), self-identified ethnicity (others, indigenous, Afro-descendant), health insurance coverage (no, yes), area of residence (urban, rural), geographic region (coast, highlands, jungle), and residence altitude (0–499 metres above sea level [m.a.s.l.], 500–1499 m.a.s.l., 1500–2999 m.a.s.l., 3000 m.a.s.l. or more).

The disability variable was constructed based on questions developed by the Washington Group [16]. These included six questions that inquired whether the respondent experienced permanent difficulties or limitations in any of the following areas: vision, hearing, speech, mobility, comprehension, or social interaction. The available response options were “yes” or “no”. A person was considered to have a disability if they answered “yes” to at least one of these questions.

### 2.6. Data Analysis

Data analysis was performed in StataNow version 18.5 SE, incorporating the complex survey design and weighting. QGIS was used to visualize regional variations in the proportion of BGT, while both StataNow version 18.5 SE and R 4.4.3 were employed to generate the figures.

Absolute and relative frequencies of the sample characteristics were calculated, and weighted proportions of BGT performance were estimated along with their 95% confidence intervals (95% CI). Differences in the proportion of BGT among population subgroups were evaluated using chi-square tests. To account for the potential for complex survey designs, the Rao-Scott correction was applied to the test of response homogeneity when analysing multiple response variables [17].

We assessed socioeconomic inequalities in BGT using concentration curves (CC) and concentration indices (CI). CCs have been widely used to model the socioeconomic gradient in health indicators [18,19]. The CC graphically represents the cumulative percentage of BGT uptake (ordinate) against the cumulative percentage of the population, ranked by socioeconomic status from the most deprived to the most affluent (abscissa). Under perfect equality—where BGT uptake is identical across all socioeconomic groups—the CC aligns with the line of equality (a diagonal from the origin to the upper right quadrant). A CC above (below) the line of equality indicates a pro-poor (pro-rich) distribution, meaning BGT uptake is higher (lower) among lower-income individuals. The degree of deviation from the line of equality reflects the magnitude of socioeconomic inequalities in BGT.

CI is a widely used measure of income-related health inequalities [19]. It quantifies the covariance between the area under the CC and the fractional rank of individuals within the income distribution, as shown in Equation (1):

(1) $$CI=\frac{2}{\mu }{cov}_{w}[y_{i}tR_{i}^{t}]$$

where i is an individual, y is the outcome variable, μ represents the mean of the outcome variable, and R is the fractional rank in the wealth index. A significant limitation of the CI for binary outcomes is its sensitivity to the mean of the outcome: as the mean of the binary outcome approaches unity (i.e., the prevalence of the outcome increases), the range of possible CI values diminishes, approaching zero. This limits its interpretability in analysing health inequalities for highly prevalent conditions. To address this limitation, Erreygers (2009) proposed the Erreygers Concentration Index (ECI) [20], which is better suited for binary health outcomes. ECI was estimated to assess the economic inequalities in BGT. The ECI was obtained by Equation (2), where y is the outcome variable, μ represents the mean of BGT in the study population, ${(a}^{max}-a^{min})$ denotes the outcome variable with limit values 0 and 1, and C(h) is the standard CI.

(2) $$ECI(y)=\left[\frac{4_{\mu }}{{(a}^{max}-a^{min})}C(h)\right]$$

For any ECI, the values range from −1 to 1, which indicates the variability and strength of the association between the variables under investigation. Positive (negative) ECI values signify a higher concentration of BGT within the wealthiest (poorest) population segments.

Inter-group differences in ECI were assessed using statistical comparisons. The z-test was applied for pairwise comparisons, while the F-test was used for multiple-group comparisons. Given the study’s large sample size, the assumption of equal variances across groups was relaxed.

To analyse the relative contribution of explanatory variables to socioeconomic inequalities in BGT, we conducted a decomposition analysis following Koolman and van Doorslaer’s framework [21]. Generalized linear models (GLMs) were used due to their robustness for decomposing inequalities in binary outcomes, outperforming methods like probit or ordinary least squares [22]. To decompose the socioeconomic inequality in BGT, Equation (3) was estimated, where ECI is the Erreygers concentration index, $\underline{x}k$ is the mean of the covariates included in the decomposition analysis, $\beta_{k}^{m}$ is the partial effect $\frac{dy}{dk}$ evaluated at the sample means, ${CI}_{k}$ is the mean of the concentration index and ${GCI}_{\varepsilon }$ is the generalized concentration index of the stochastic term of error.

(3) $$ECI=4*{[\Sigma }_{k}(\beta_{k}^{m}\underline{x}k){CI}_{k}]+{GCI}_{\varepsilon }$$

Equation (3) demonstrates that an explanatory variable contributes to socioeconomic inequalities in BGT exposure only if it exhibits two key characteristics: (1) a statistically significant correlation with BGT levels and (2) an unequal distribution across socioeconomic strata, as measured by the wealth index. The magnitude of an explanatory variable’s contribution to inequality is determined by the interplay of two factors: the absolute value of its partial effect on BGT and the degree of its uneven distribution across the wealth gradient. A positive partial effect signifies that the explanatory variable exacerbates existing inequalities in BGT exposure, while a negative partial effect indicates that it mitigates such inequalities.

### 2.7. Ethical Considerations

For this study, ethical approval was not required since publicly available, properly anonymised data were used. The data utilised are available on the INEI website https://proyectos.inei.gob.pe/microdatos/ (accessed on 12 December 2024).

### 2.8. Reporting Standards

This manuscript adheres to the STROBE (Strengthening the Reporting of Observational Studies in Epidemiology) guidelines for reporting cross-sectional studies [23]. The STROBE checklist is available in the Supplementary Materials.

## 3. Results

A total of 9499 participants were included, with 53.6% being women. Most were aged 35–49 years (59.4%), and 42.2% had completed secondary education. Only 1.3% reported having a disability. More details on the participants’ characteristics can be seen in Table 1.

Women had a higher BGT uptake (34.1%; 95% CI: 31.9–36.3) than men (29.6%; 95% CI: 27.5–31.8; *p* = 0.005). Regarding educational level, individuals with higher education exhibited the highest testing proportion (42.5%; 95% CI: 39.8–45.3; *p* < 0.001). Geographically, BGT uptake was higher in urban areas (34.3%; 95% CI: 32.6–36.1) and in lower-altitude regions (0–499 m.a.s.l.: 34.7%; 95% CI: 32.7–36.8) (Table 2).

**Figure 1:**
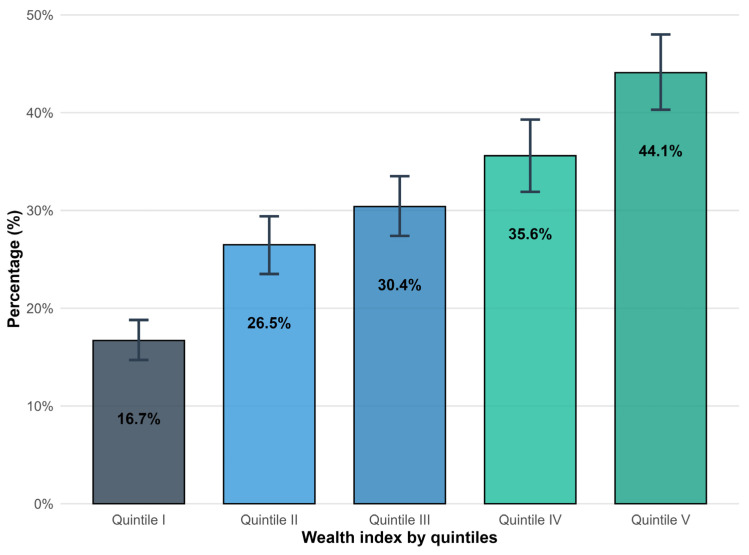
Proportion of Peruvian adults undergoing blood glucose testing by wealth index quintiles.

Figure 1 shows the proportion of individuals who underwent a BGT in the last year according to wealth quintile. An increasing gradient is observed in the proportion of people reporting that they had a BGT, with a 27-percentage point difference between quintile I and quintile V (*p* < 0.001).

**Figure 2:**
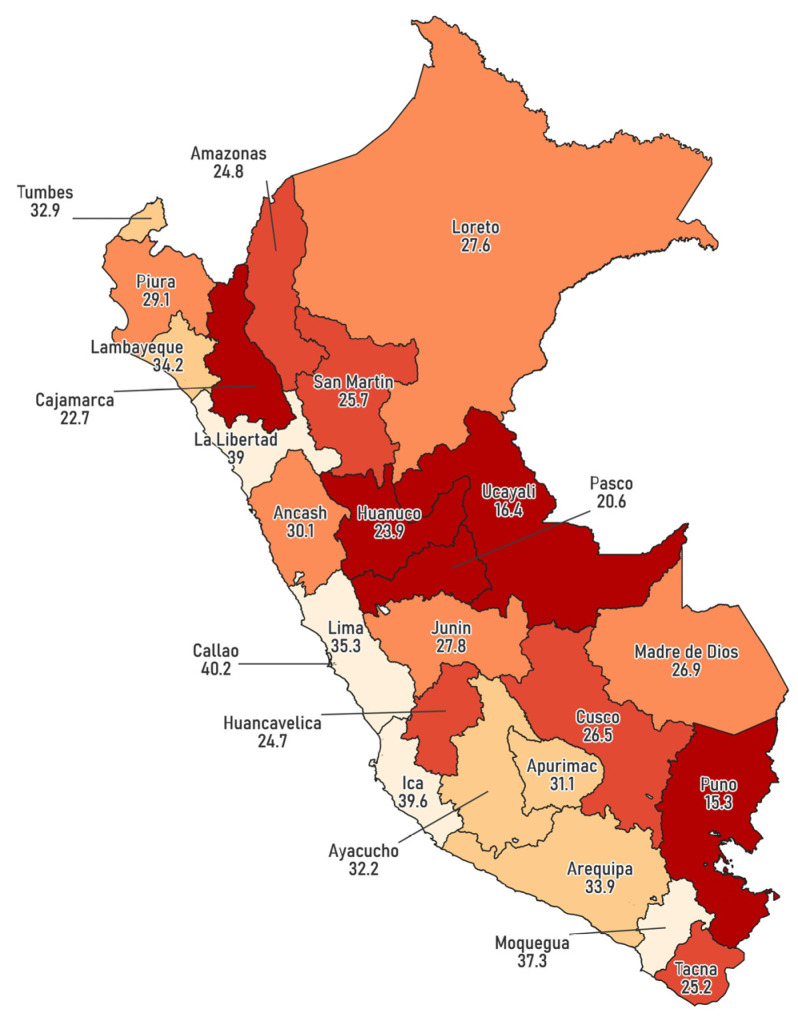
Regional distribution of blood glucose testing among adults in Peru, 2023.

The regions with the highest proportions include Callao (40.2%), Ica (39.6%), Moquegua (37.3%), and Lima (35.3%), while the lowest values were recorded in Ucayali (16.4%) and Puno (15.3%) (Figure 2).

**Figure 3:**
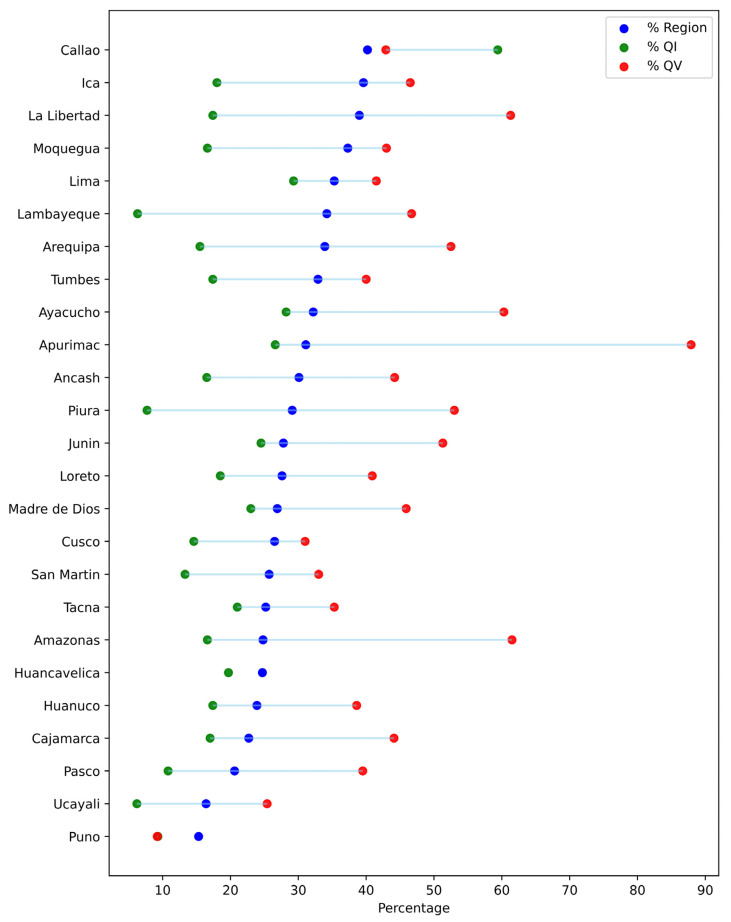
Comparison of blood glucose testing proportions by region, lowest quintile (QI), and highest quintile (QV) in Peru, 2023.

In addition, marked variability is observed both between regions and within quintiles (Figure 3). In regions such as Lima and Tacna, the differences between quintiles are less pronounced, whereas in regions such as Amazonas and Apurímac, the intra-quintile differences are wider.

**Figure 4:**
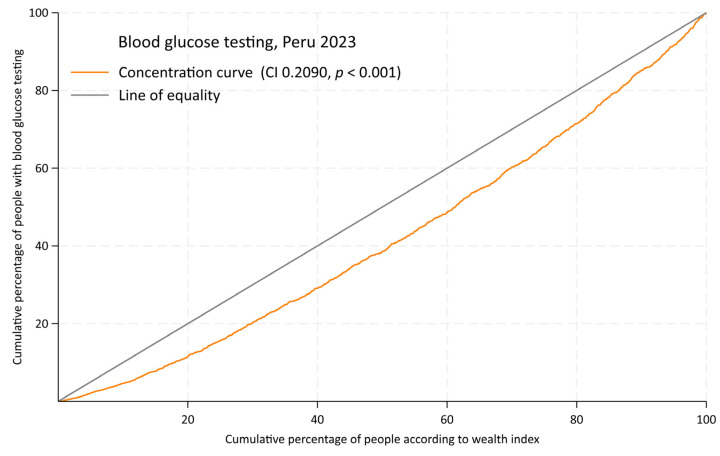
Concentration curve for blood glucose testing in Peru, 2023. CI: concentration index.

The CC for BGT shows a pro-rich concentration (Figure 4), with a CI of 0.2090 (*p* < 0.001). This indicates that individuals with a higher wealth index have significantly greater access to BGT than those with a lower wealth index.

**Figure 5:**
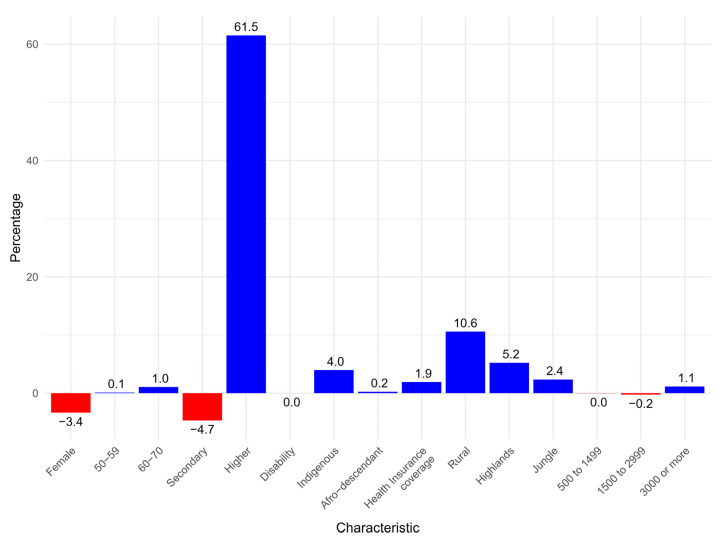
Decomposition of socioeconomic inequalities in blood glucose testing in Peru, 2023.

Decomposition of socioeconomic inequalities revealed that higher education contributed 61.5% to pro-rich socioeconomic inequalities, followed by rural residence, which contributed 10.6% to these inequalities. Conversely, secondary education and female gender reduced pro-rich socioeconomic inequalities by 4.7% and 3.4%, respectively (Figure 5).

## 4. Discussion

This study shows significant socioeconomic and geographic inequalities in BGT uptake among overweight and obese adults in Peru. Rural populations, people at higher altitudes, and those in the lowest wealth quintiles reported lower testing prevalence. The decomposition of socioeconomic inequalities showed that higher education and residence in rural areas were the primary contributors to pro-rich inequalities in BGT uptake. In contrast, secondary education and female gender were found to contribute to a reduction in these inequalities.

Urban residents had a higher prevalence of BGT than their rural counterparts, reflecting persistent urban-rural inequalities in healthcare access commonly observed in LMICs [24,25]. Similarly, individuals at lower altitudes exhibited higher testing rates than those in high-altitude regions, likely due to challenges related to healthcare infrastructure and physical accessibility [25,26,27]. At the regional level, substantial variability in BGT uptake was observed, likely influenced by differences in urbanization and healthcare infrastructure [28]. Remote areas such as Puno (Andes) and Ucayali (Amazon) had the lowest testing rates, potentially due to geographic isolation and limited healthcare access [29]. Additionally, Apurímac and Amazonas showed the largest socioeconomic inequalities in BGT uptake between the highest and lowest wealth quintiles, likely driven by structural barriers and socioeconomic inequalities. Both regions face significant healthcare infrastructure challenges, high poverty levels, and low educational attainment, disproportionately affecting the poorest groups [28]. These findings underscore the need for regionally tailored interventions that expand healthcare access in remote areas through targeted investments and community-based approaches.

Our findings showed a pronounced inequality across wealth quintiles. A total of 27 percent points for BGT between the highest and lowest wealth quintile was observed. This gradient highlights the persistent role of socioeconomic status in determining access to preventive health services [30], particularly in LMICs where payment for healthcare is mainly out of the patient’s pocket [25]. Furthermore, educational level also showed inequalities, with those with higher education more frequently undergoing a BGT than their less educated peers. Lack of education has been found to limit healthcare access in all settings, as it reduces awareness of preventive care and perceived need [25].

A pro-rich concentration in BGT uptake was observed, highlighting significant socioeconomic inequalities. Decomposition analysis revealed that higher education contributed the most to inequality (around 60%), consistent with findings from Kerala, India, where education was a key factor in BGT uptake [31]. The role of education in inequality appears to stem from differences in health knowledge and perceived need, a pattern observed across both high- and low-income settings [31]. This is further supported by evidence showing that individuals with higher education have nearly twice the odds of health-seeking behaviour compared to their less educated peers (Odds Ratio [OR] = 1.87, 95% CI: 1.38–2.55) [32]. Rural residence was the second-largest contributor to inequality, exacerbating the pro-rich distribution of BGT uptake. This aligns with studies from other settings, such as high-altitude regions in Peru [26]. Evidence shows that geographic remoteness and inadequate healthcare infrastructure limit access to preventive services [25,26]. Rural residents in Peru face additional barriers, including fewer healthcare facilities, a lower density of healthcare professionals, and higher transportation costs [29], underscoring the need for geographically targeted interventions.

Conversely, gender contributed to reducing pro-rich inequalities in BGT, consistent with global trends showing that women seek preventive care about twice as often as men [33,34], particularly in LMICs [25]. Several reasons may explain these findings. Women are more likely than men to undergo preventive services such as blood pressure and cholesterol screening, even after adjusting for several factors including socioeconomic status, insurance coverage, and perceived health [35,36]. Similarly, it has been reported that women have more frequent ambulatory preventive care visits [35]. Together, these factors may increase their exposure to preventive interventions. In addition, women exhibit greater interoceptive awareness, i.e., the ability to perceive internal bodily sensations, which has been linked to more proactive health-seeking behaviour [37,38]. Conversely, men are more likely to engage in risky health behaviours, such as smoking and excessive alcohol consumption, which may further deter engagement with preventive care [33]. Furthermore, traditional masculine norms that emphasize self-reliance and emotional restraint may discourage men from engaging with preventive health services [33,39]. These combined behavioural and structural dynamics likely contribute to the narrowing of inequalities in BGT uptake observed among women.

### Strengths and Limitations

This study has some limitations to note. The use of self-reported data for BGT uptake introduces the possibility of recall bias. However, the question used in the ENDES follows the time frame and action structure proposed by the WHO STEPS approach, ensuring standardization and comparability in the way the information was collected [40]. The exclusion of people with diagnosed diabetes relied on self-reported information rather than laboratory-confirmed data. This limitation may have led to the inadvertent inclusion of individuals with undiagnosed diabetes who reported undergoing glucose testing, introducing a non-differential misclassification bias that could have underestimated the true inequalities among at-risk populations. The altitude of residence was determined at the cluster level rather than the specific household location, which may have introduced inaccuracies in classifying altitude at the individual level. The analysis was performed on a subpopulation of the ENDES dataset, comprising adults aged 35–70 years with overweight or obesity. While this focus aligns with the study’s objective, it likely reduced the sample size available for stratified analyses by wealth index and at the regional level, potentially affecting the precision of prevalence estimates. Finally, the analysis was limited by small sample sizes when stratifying simultaneously by ethnicity and sex, which prevented reliable subgroup estimates. Although ENDES captures self-reported ethnicity, a valid approach in the Peruvian context [41], the sample sizes for minority groups were relatively small. This limited our ability to explore differences in access to preventive care among specific ethnic and sex groups. Additionally, potential misclassification in self-reported ethnicity cannot be ruled out, as social desirability and contextual factors may influence individual responses [41].

On the other hand, several strengths are worth highlighting. This study utilised a population-based database, enabling inferences at both national and regional levels. While the outcome variable relied on self-reported data, the question used to identify individuals undergoing BGT adhered to the WHO STEPS framework for this type of assessment. Furthermore, self-reported measures in diabetes have been shown to be reliable [42,43].

## 5. Conclusions

This study highlights significant socioeconomic and geographic inequalities in BGT among overweight and obese adults in Peru. Uptake is lower in rural areas, higher altitudes, and the lowest wealth quintiles. The large gap between the highest and lowest wealth quintiles underscores the critical role of socioeconomic status in access to preventive care. These findings call for targeted interventions, such as expanded healthcare infrastructure and community-based screening programmes, to address inequalities and reduce the burden of undiagnosed diabetes in high-risk populations.

## Figures and Tables

**Table 1:** Sociodemographic characteristics of the Peruvian adults included in the study, Peru, 2023.

Characteristic	*n*	% Weighted
Gender		
Male	4007	46.4
Female	5492	53.6
Age groups (years)		
35–49	6472	59.4
50–59	2116	29.0
60–70	911	11.6
Education level		
Up to primary	2773	22.5
Secondary	3882	42.2
Higher	2844	35.3
Disability		
No	9395	98.7
Yes	104	1.3
Self-identified ethnicity		
Others	4962	60.6
Indigenous	3551	28.1
Afro-descendant	986	11.3
Health insurance coverage		
No	1201	15.3
Yes	8298	84.7
Area of residence		
Urban	6483	85.1
Rural	3016	14.9
Geographic region		
Coast	4063	65.8
Highlands	3240	22.5
Jungle	2196	11.7
Residence altitude (m.a.s.l.)		
0 to 499	4903	68.6
500 to 1499	1135	7.9
1500 to 2999	1494	10.7
3000 or more	1967	12.8

The weighting factor and sample specifications of ENDES were included. m.a.s.l.: metres above sea level.

**Table 2:** Proportions of blood glucose testing by sociodemographic characteristics, Peru, 2023.

	Blood Glucose Testing				
	No		Yes		
Characteristic	% Weighted	(95% CI)	% Weighted	(95% CI)	*p*-Value
Gender					
Male	70.4	(68.2–72.5)	29.6	(27.5–31.8)	0.005
Female	65.9	(63.7–68.1)	34.1	(31.9–36.3)	
Age groups (years)					
35–49	69.9	(68.0–71.6)	30.1	(28.4–32.0)	0.001
50–59	67.4	(64.0–70.7)	32.6	(29.3–36.0)	
60–70	59.6	(54.6–64.5)	40.4	(35.5–45.4)	
Education level					
Up to primary	77.4	(74.8–79.9)	22.6	(20.1–25.2)	<0.001
Secondary	71.7	(69.3–74.0)	28.3	(26.0–30.7)	
Higher	57.5	(54.7–60.2)	42.5	(39.8–45.3)	
Disability					
No	68.0	(66.4–69.5)	32.0	(30.5–33.6)	0.910
Yes	68.7	(54.2–80.4)	31.3	(19.6–45.8)	
Self-identified ethnicity					
Others	64.8	(62.7–66.9)	35.2	(33.1–37.3)	<0.001
Indigenous	73.3	(70.7–75.7)	26.7	(24.3–29.3)	
Afro-descendant	71.9	(67.8–75.6)	28.1	(24.4–32.2)	
Health insurance coverage					
No	83.0	(79.1–86.3)	17.0	(13.7–20.9)	<0.001
Yes	65.3	(63.6–66.9)	34.7	(33.1–36.4)	
Area of residence					
Urban	65.7	(63.9–67.4)	34.3	(32.6–36.1)	<0.001
Rural	80.9	(79.1–82.6)	19.1	(17.4–20.9)	
Geographic region					
Coast	64.7	(62.5–66.8)	35.3	(33.2–37.5)	<0.001
Highlands	74.4	(72.2–76.5)	25.6	(23.5–27.8)	
Jungle	74.2	(71.9–76.3)	25.8	(23.7–28.1)	
Residence altitude (m.a.s.l.)					
0 to 499	65.3	(63.2–67.3)	34.7	(32.7–36.8)	<0.001
500 to 1499	73.2	(69.2–76.8)	26.8	(23.2–30.8)	
1500 to 2999	71.1	(67.8–74.2)	28.9	(25.8–32.2)	
3000 or more	76.8	(73.9–79.4)	23.2	(20.6–26.1)	

Data are displayed as weighted % of the row unless indicated otherwise. The weighting factor and sample specifications of ENDES were included. The *p*-value was estimated using the chi-square test with Rao–Scott adjustment. CI: confidence interval. m.a.s.l.: metres above sea level.

## Data Availability

The data sheet, questionnaire, dictionary, manuals, and databases are publicly available on the ENDES website https://proyectos.inei.gob.pe/endes/ (accessed on 12 December 2024) and INEI https://proyectos.inei.gob.pe/microdatos/ (accessed on 12 December 2024).

## Supplementary Materials

The following supporting information can be downloaded at: https://www.mdpi.com/article/10.3390/epidemiologia6020020/s1, STROBE (Strengthening the Reporting of Observational Studies in Epidemiology) guidelines for reporting cross-sectional studies.
